# Clinic Design as Placebo—Using Design to Promote Healing and Support Treatments

**DOI:** 10.3390/bs7040077

**Published:** 2017-11-09

**Authors:** Jonas Rehn, Kai Schuster

**Affiliations:** Darmstadt University of Applied Sciences, 64295 Darmstadt, Germany

**Keywords:** design research, empirical design research, placebo effect, concept priming, health behavior, embodiment, self-efficacy, salutogenic design, psycho-socially supportive design, evidence-based design

## Abstract

Analogously to the medical placebo effect, people seem to anticipate the quality of treatments based on external stimuli. In order to gain insights on the effect the built environment can have on a person’s judgments and behavior with a particular focus on health related issues, a quantitative survey (*N* = 851) with four groups before and after the renovation of a rehabilitation clinic has been conducted. In line with an overall modernization of the clinic, the entrance, the lobby, and some patient rooms have been changed. In the lobby, a service counter and coffee bar have been added as well as light colors and new flooring material to achieve a more modern and clean atmosphere in the sense of aesthetical appearance of the space. The outcome revealed that patients rate the intention to change their health behavior as well as the quality of food or significantly higher in a modernized clinic. These differences cannot be directly attributed solely to the changes in the building. Analogously to the medical placebo, an effect referred to as design placebo effect is, therefore, proposed to explain improved ratings of aspects that have not directly been changed due to the intervention. Other significant effects are attributable to winter and summer climate. During summer time, ratings for waiting area, atmosphere, patient rooms, as well as for staff were significantly higher. It is, therefore, assumed that aesthetic attributes, such as architectural design, or friendliness of the weather, exert their effects as perceptual placebos that directly influence judgment outcomes and behavioral intentions. Further research is needed to match certain design and general environmental features to their effects on patients and investigate their effect strength.

## 1. Introduction

In the therapeutic field medical placebos are defined as “substances that are administered in the guise of active drugs but that do not in fact have the pharmacological properties attributed to them” [1] (p. 1189). Their effects are therefore attributed to psychological processes, namely classical conditioning and response expectancy [1,2]. As evidence in the field of behavioral economics and marketing research points out, this effect of response expectancy applies to non-therapeutic contexts as well [3]. Attributes such as pricing or general appearance seem to have an effect on user’s expectancy of the quality and efficacy of a product. While this phenomenon—knowingly or unknowingly—has been used in the retail business for decades [4], it seems to be neglected in the design of therapeutic contexts like hospitals or rehabilitation clinics.

Following the research and practice from consumer and marketing research, it can be assumed that the mere aesthetic quality of the built environment (e.g., the clinic design) can have a significant effect on the patients’ expectancy of their medical treatment and consequently their own health behavior (e.g., motivation and self-efficacy) [5,6]. In fact, the current design paradigms underline the therapeutic effects of the built environment [7,8,9], suggesting an evidence-based design approach [10,11,12] in order to promote health through design [12,13]. Notably, Ulrich’s idea of a psychosocially supportive design [14] and the salutogenic design proposed by Dilani [15] criticize modern health buildings as being “starkly institutional, stressful, and detrimental to care quality” [9] (p. 49).

Therefore, the renovation of a rehabilitation clinic in Bad Kreuznach, Germany has been used as a natural intervention to investigate the effect the new clinic design has on patient’s judgment and behavior. Comparable studies showed a link between physical environment and treatment response depending on patient’s aesthetic preferences [16]. It has been assumed that the new clinic design with its brighter entry hall and a more modern look makes the patients expect better treatment and to find better staff in the new clinic setting. According to the placebo effect, this leads to an overall better judgment on these items. 

## 2. Materials and Methods

**Figure 1:**
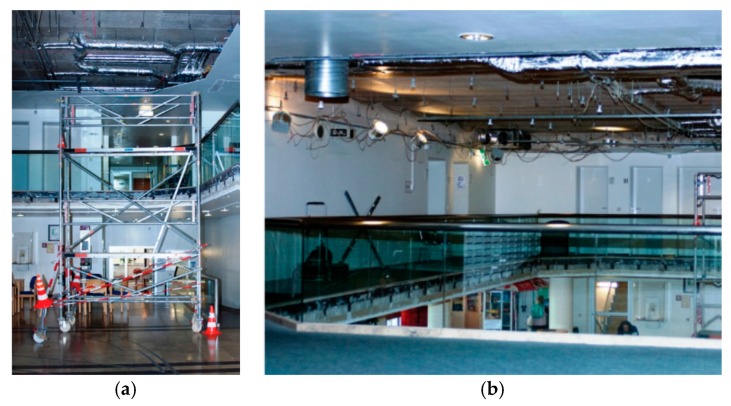
Situation during phase 1 (pre-group): (**a**) Ground floor with dark wooden flooring material; (**b**) First floor with worn-off blue carpet. (Picture credits: © Matthias Broer, Brigitte Pfeif, Hildegard Metz, 2013).

**Figure 2:**
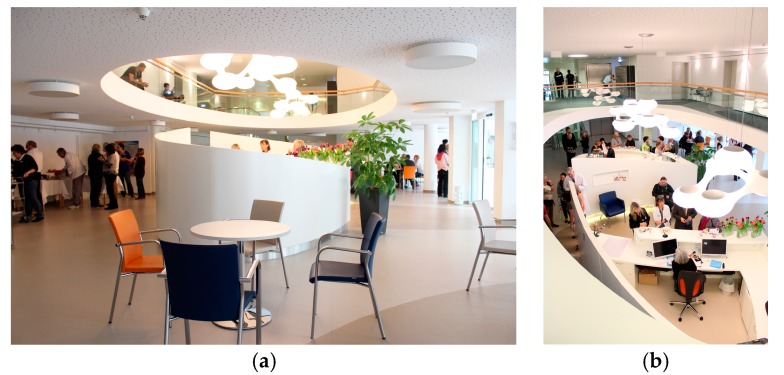
Situation after intervention (phase 2–4; post-group): (**a**) Ground floor with service counter in the center. Light colors and a variety of flexible seating arrangements create a more appealing atmosphere; (**b**) View onto service counter from first floor. Modern light sculptures and organic shapes support a friendly and welcoming appearance. (Picture credits: © Matthias Broer, Brigitte Pfeif, Hildegard Metz, 2013).

The setting of this study is the rehabilitation clinic building of the Acura Clinic in Bad Kreuznach, Germany. Due to a general modernization program, several architectural and design aspects of the building have been changed. The major intervention took place in the lobby, which is located directly at the entrance of the building. Here, the circulation to the building has been moved from a main road to a side road. Furthermore, a centralized service counter and a coffee bar have been placed at the center of the lobby, leading to an easier orientation in the room. A new light concept including bigger windows and more artificial light have been implemented to the new room as well. The former dark blue worn-off carpet and dark brown floor coating have been replaced by a light grey PVC floor coating (see Figure 1 and Figure 2). In addition to the changes in the lobby, some of the patient rooms have been modernized as well. The new patient rooms included bright furniture and color elements at the walls, leading to an overall friendly appearance. Apart from the lobby and some of the patient rooms, no other part of the clinic (e.g., therapy rooms or training facilities) has been changed in any way. Further, the staff as well as the dining facilities remained the same as before the renovation of the clinic. 

In order to measure the effect of these changes on the judgment and behavior of the patients, a quantitative survey (*N* = 851) has been performed in four different phases:

- Phase 1: October 2013–December 2013 (*N*_1_ = 211) (before/during renovation process = baseline)
- Phase 2: April 2014–August 2014 (*N*_2_ = 212) (after intervention; post-group)
- Phase 3: October 2014–December 2014 (*N*_3_ = 215) (after intervention; post-group)
- Phase 4: October 2015–December 2015 (*N*_4_ = 212) (after intervention; post-group)

To measure the effect of the intervention a questionnaire with 22 items has been developed, including 15 items on a 5-point Likert-scale, 4 items as open questions, and 3 nominal items (for a PDF of the German questionnaire see Supplementary Materials). Apart from sociodemographic questions, the survey focused mainly on the satisfaction with staff, treatment, food, room, and building qualities as well as health-related intentions. Rooms that have been renovated (patient room and lobby) and rooms and facilities that remained the same have been rated separately, allowing the measurement of correlations between the two. 

Since it is a standard procedure in the clinic to ask the patients to rate the clinic at the time of departure, the survey could easily be embedded in the daily routine of the staff. Blending in with the rating questionnaire of the clinic, the survey did not stand out as an artificial research activity which otherwise might have operated as a confounder, thus allowing a higher degree of external validity. All patients were asked to participate in the survey. The questionnaires were filled out and handed in anonymously. 

In reference to the medical placebo effect, the existence of a similar design placebo effect is proposed. Design placebos are architectural and design elements that suggest certain attributes (or in the presented case improvements) that are not existing. However, these elements lead to a similar effect as if the attributes existed. To investigate this effect, the following hypotheses were made:

H1: Architectural and design features influence factors that are not directly linked to the healing process but have some impact on it.

Based on this hypothesis the following sub-hypotheses emerged:

H1.1: The design of the lobby influences the evaluation of social interactions.

H1.2: The design of the lobby influences the evaluation of the therapy and the medical treatment.

H1.3: The design of the lobby influences the evaluation of other rooms in the building, although their condition equal during the study.

H1.4: The design of the lobby influences the evaluation of the medical staff.

H1.5: The design of the lobby influences the evaluation of the food that is served during rehabilitation.

In addition to the effect architectural and design elements have on the judgment of the patients, it is a focus point of this study to investigate their effect on health behavior. Due to methodic reasons it was not possible to track the actual health behavior of the participants. In order to gain knowledge of possible health behavior changes, the intention to change one’s health behavior has been asked during the questionnaire. According to current paradigms in health behavior research such as the transtheoretical model, intention is a major requirement for behavioral change to occur [17]. Hence the hypothesis:

H2: The design of the lobby influences health related behavioral intentions.

Since the survey took place both during summer and winter time, the effect of seasonal conditions could be investigated as well. With regards to the above mentioned items, due to research findings on the effect of seasonal conditions on mood and cognitive performance [18] more positive ratings during the summer season have been anticipated.

H3: Seasonal conditions influence the evaluation of the clinic and clinic related aspects.

Some patients (*N* = 38) stayed in modernized patient rooms, allowing the effect of the patient room design to be observed as well.

H4: The design of the patient room influences the evaluation of the clinic and clinic related aspects.

## 3. Results

### 3.1. Descriptive Data

In total, 851 people participated in the study. In the first phase before and during the intervention, 211 patients and in the three post-intervention phases 640 patients took part in the survey. 

### 3.2. Influence of Seasonal Conditions

While phase 2 (summer group) was conducted during summer time (April 2014–August 2014), phase 3 and 4 (winter group) took place in winter time (October 2014–December 2014 and October 2015–December 2015). Since all three phases belong to the post-intervention group, differences between phase 2 and phase 3 and 4 could not be attributed to changes in the building.

Using a multiple analysis of variance (MANOVA), significant differences between summer and winter group could be found for the evaluation of staff (*p* = 0.013), patient room (*p* = 0.029), waiting area (*p* = 0.001), atmosphere (*p* = 0.002), food (*p* = 0.019), and the stay in general (*p* = 0.006). All these items ranked higher in summer than in winter times. 

The results can be explained by the fact that seasonal conditions (e.g., more sun exposure, higher temperature in summer time) and the general activity options that are related to it (e.g., festivals in the city, possibility to have a longer walk, outdoor sports, etc.) lead to a higher degree of overall satisfaction by the patients [18]. This again had a positive influence on the rehabilitation experience. Therefore, the H0_3_-hypothesis can be rejected and the H3-hypothesis is accepted. With regards to the above mentioned design placebo effect, seasonal conditions exert their effect rather as a prime than placebos. 

### 3.3. Influence of the Architectural Intervention on Patient’s Judgment and Behavior

As presented in Section 3.2, seasonal conditions are found to impact the evaluation of the above mentioned items. Thus, in order to investigate the effect, the architectural and design intervention have on the judgment and behavior of the patients the summer group can be seen as a confounding variable and must be excluded from further analysis.

Using an analysis of variance (ANOVA), a significant difference of variances (*p* = 0.010) between phase 1 and phase 4 has been identified. A post-hoc *t*-test (α = 5%) revealed better ratings for waiting areas (*p* < 0.001), atmosphere (*p* < 0.001), and overall rehabilitation experience (*p* = 0.010). However, these findings do not contribute to what above has been defined as design placebo, since all three aspects have been directly changed by the intervention itself. 

In addition to that, the *t*-test confirmed significantly better ratings for training rooms (*p* = 0.045) and food (*p* < 0.001) and a tendency towards significance for the therapy rooms (*p* = 0.057). These differences between pre- and post-interventional survey cannot be explained by the architectural and design changes that were made during the intervention, since training and therapy rooms as well as food and beverages have not been changed. It is, therefore, assumed that the intervention acts as a design placebo that leads to higher expectations regarding the overall quality of the building, staff, and related aspects (e.g., food and beverages). As described by the idea of response expectancy [2] these higher expectations might result in more positive experiences with the above mentioned items. 

Therefore, the H0_1_-hypothesis can be rejected and the H1-hypothesis is accepted.

### 3.4. Influence of the Architectural Intervention on Patient’s Health Behavior Intention

**Figure 3:**
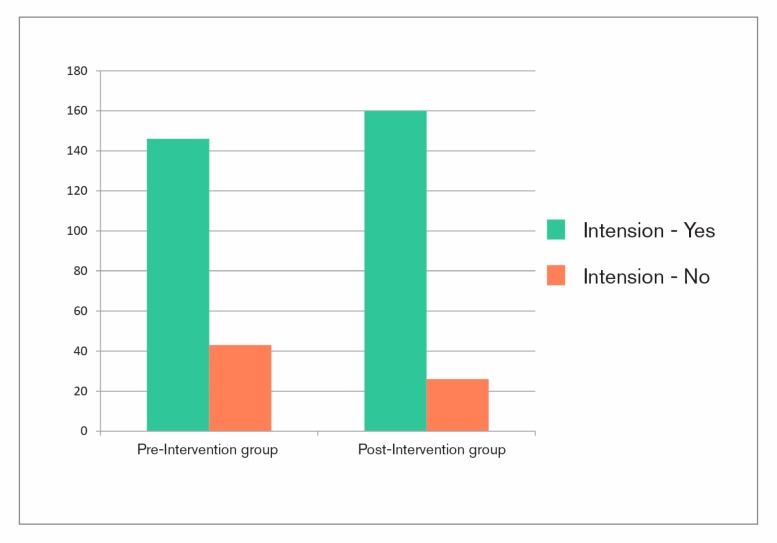
Distribution of positive and negative remarks concerning the intention to change one’s health behavior due to rehabilitative treatment.

**Figure 4:**
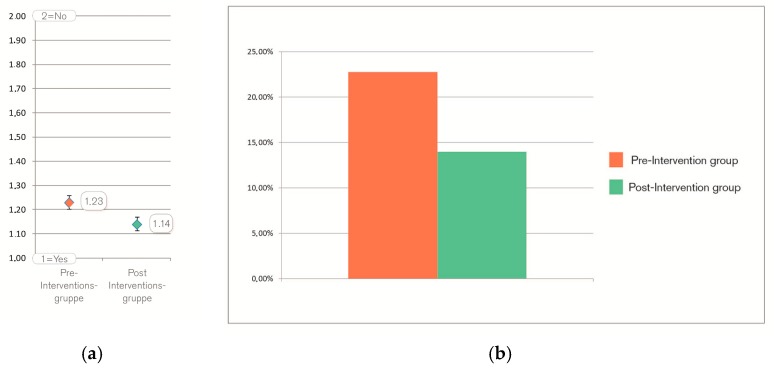
On average, patients rated on the dichotomous item in the pre-group 1.23 pts and in the post-group 1.14 pts (with 2 pts. being “yes” and 1 pt. being “no”), which is a difference of 9 % of the total item range (**a**); The relative prevalence of patients with “no intentions” changed between pre- and post-group from 22.75% to 13.98% (**b**), which is a total reduction of relative prevalence of 38.55%.

As mentioned before, due to methodical reasons it was not possible to track the difference in health behavior of both treatment groups. Therefore, item 10 of the questionnaire referred to the intention to change one’s health behavior: “Do you think, your stay at the clinic will change your daily health behavior persistently?” (translated from German). Patients in the post-intervention group rated significantly more in favor of this question (*p* = 0.028) (see Table 1 and Figure 3). On average, the difference between both groups is 9% (see Figure 4a).

The relative prevalence of patients with “no intentions” changed between pre- and post-group from 22.75% to 13.98%, which is a total reduction of the relative prevalence of 38.55% (see Figure 4b). 

Thus, the renovated and, therefore, aesthetically improved design of the lobby seems to make the clinic appear more effective in the sense that patients are more committed to the overall health advices they obtain during their stay [19,20]. As a consequence, the design of the lobby seems to influence health related behavioral intentions. Therefore, the H0_2_-hypothesis can be rejected and the H2-hypothesis is accepted.

### 3.5. Influence of the Design of the Patient Room on the Evaluation of the Clinic and Clinic Related Aspects

**Figure 5:**
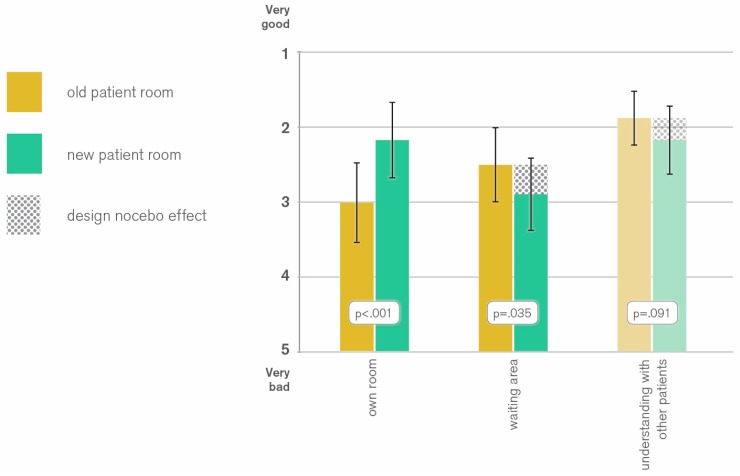
*t*-Test analysis of effects of old and new patient rooms on various evaluation aspects showing a design nocebo effect.

As expected, patients rated their own patient rooms higher, if they stayed in a renovated room (2.99 vs. 2.16 pts; *p* < 0.001) (see Figure 5). This cannot be regarded as a design placebo effect, since it only proves that the intervention has improved the appearance of the patient room.

However, patients in new patient rooms showed a tendency to significance for lower ratings of physicians (1.97 vs. 2.25 pts; *p* = 0.089) and social understanding between patients (1.87 vs. 2.16 pts; *p* = 0.091). Further, they evaluated the waiting area significantly lower (2.49 vs. 2.88 pts; *p* = 0.035). 

The poorer evaluation of social understanding and waiting area can partly be explained by the assumption that patients spend more time in their rooms if they are renovated. This leads to lesser social interactions with other patients and lower attribution of value in the use of the waiting area. On the other hand, one could argue that patients staying in a renovated patient room have a higher baseline for the evaluation of other aspects of the clinic. As opposed to patients in old patient rooms, to the ones in renovated rooms the waiting area looks less attractive in comparison to their own room. This negative side effect of the aesthetic improvement of the patient room is in this study is referred to as a ‘design nocebo’ effect, analogously to the ‘medical nocebo’, which is known as negative side effects of substances that actually have no pharmacological effect [21]. As a consequence, the lower ratings for social understanding can be classed with the design nocebo effect as well, since it can be regarded as an indirect negative consequence of an aesthetical improvement.

Therefore, although with a negative sign, the H0_4_-hypothesis can be rejected and the H4-hypothesis is accepted.

### 3.6. Influence of the Design of the Patient Room on the Evaluation of the Clinic and Clinic Related Aspects

**Figure 6:**
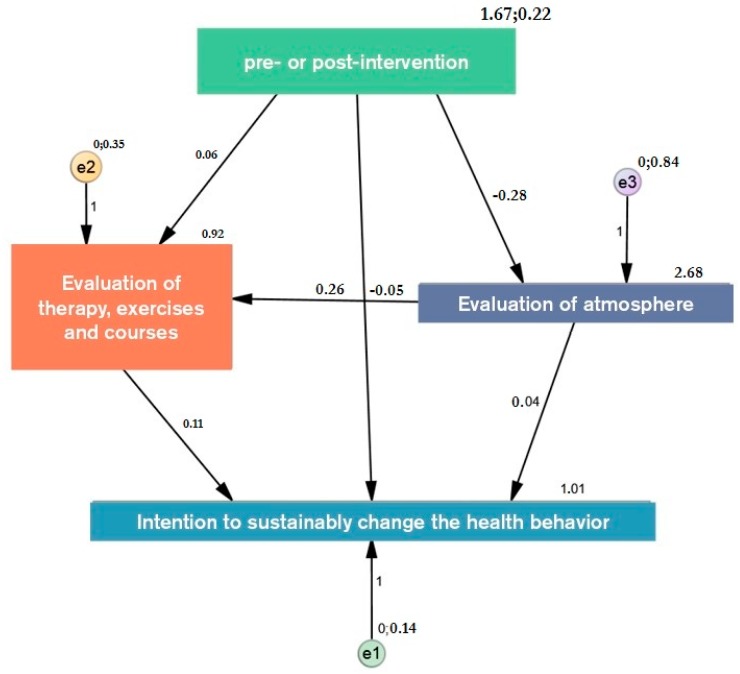
Path analysis of first and last phase.

In order to identify causality of the above presented results, a path analysis has been conducted (*N*_1_ = 204; *N*_2_ = 187) (see Figure 6). The main focus of this path analysis is to investigate the relationship between intervention and intention to change health behavior. The total structure of the model shows good values with a normed fit index of 1 and a comparative fit index of 1. Although, the RMSEA of 0.147 indicates that the model represents the reality not as complex as necessary, it gives rise to the assumption that the hypothesized relations between the various items exists. Further research is needed to specify these findings. 

In addition to a direct link between intervention and intention, the path analysis indicates a tendency to significance that the effect is mediated by other factors (*p* = 0.095) (see Table 2). Further, the results show that the changes of the built environment influence the evaluation of the atmosphere (−0.28; *p* < 0.001), which in turn influences the evaluation of therapy and courses (0.26; *p* 0.001). The presented effect on the intention to change one’s health behavior (0.11; *p* < 0.001) is, therefore, partly caused by the changes of the built environment. Thus, atmosphere and therapy work as mediators between intervention and health related intention. Furthermore, atmosphere exerts a direct effect on the intention by 0.04 (*p* = 0.021). The analysis gives rise to the assumption that the design and architectural elements, as well as other aesthetic attributes of the environment, significantly influence the health behavior of people in this context. 

## 4. Discussion

The results confirm that apart from other factors such as seasonal aspects, changes of the design of the built environment have an influence on aspects that are not directly linked to the environment itself. Based on the medical placebo effect and according to the herewith presented design placebo effect, it can be assumed that the design of the built environment as well as particular design features in this environment evoke expectations that influence the actual experience. As described above, this might have an impact on a person’s intention and thus behavioral decisions. 

**Figure 7:**
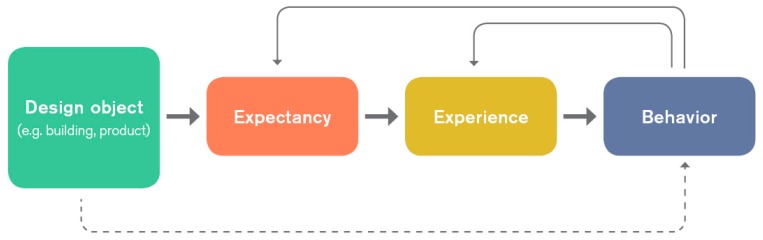
Model of the design placebo effect and its behavioral impact.

As shown in Figure 7, this can create a circular effect in which design attributes influence experiences and behaviors that again influence certain expectations, experiences, and behaviors. 

However, due to the highly complex field of the study with a big diversity of patients and corresponding symptoms, confounding variables such as social dynamics or characteristics of certain illnesses might have an influence on the results. This applies particularly to the effect the intervention has on the staff. Their mood and motivation might be influenced by the environment as well, which leads to corresponding interactions with the patients.

Furthermore, since several design features have been changed during the intervention (flooring material, additional counters in the lobby, change of circulation, etc.) the found effects cannot be attributed to a single design feature. Therefore, further research should investigate in more detail the existence of an effect that can be allocated to particular single design and architectural features (e.g., in a more controlled environment such as a laboratory experiment). In addition to that, further research is required in order to confirm these effects for other user groups (e.g., non-patient environments). 

Apart from that, more research is needed to understand how certain expectations are being formed. Individual experiences as well as cultural aspects might have an impact on the way people perceive certain design elements and styles and thus influence the design placebo effect accordingly. Furthermore, the results give rise to questions of how to prevent unwanted effects of styles by particular user groups. This applies in particular to the differences in taste between designers/architects and users i.e., patients [22]. With the effect being complex, we recommend integrating end-users in the research and design process in order to identify how certain styles and design attributes are being perceived and to which expectations they lead. User-needs-analysis, as well as post-occupancy-evaluations such as Sandal et al. [16], are recommended to investigate the effects design decisions have on users especially in the therapeutic context. For operators of clinics, results of these studies might be financially as well as therapeutically helpful.

## 5. Conclusions

The design of therapeutic contexts influences the judgment and behavior of patients in this context in a wider sense than the immediate environment. This direct link can be seen as a design placebo effect and in its negative consequence as a design nocebo effect. Designers and architects should, therefore, be aware of the complex effect their work has on the overall appearance of not only the built environment but also the people working in it and the procedures and services that are offered in this context. As a result of this study, we recommended changing the design of existing clinics at once rather than in steps, since the aesthetical improvement of single elements of a complex set can have a negative influence on the appearance of other (older) elements of this setting. 

Apart from the need for more research on the particular way this design placebo effect works, from an architectural and design research point of view, it is necessary to investigate the creative possibilities of these results. The study raises questions on the way certain aesthetic elements and styles lead to which perceived effect. Furthermore, it is recommended that design researchers investigate how far aspects such as seasonal conditions can be used in the most effective way in order to improve the overall therapeutic experience. Furthermore, since the design placebo effect is based on the mere appearance of complex aspects—such as professionalism and effectiveness—more design research is needed to investigate which design features support the aesthetic perception of these aspects.

From an ethical point of view, it is also of crucial importance to understand the moral implications the design placebo effect has [23]. On the one hand, the effect might lead to better therapeutic outcomes due to higher degrees of commitment, motivation, and satisfaction. On the other hand, the effect is based on the mere appearance despite the actual existence of the perceived attributes. Ironically, a clinic with mediocre quality of treatment might have a superior therapeutic outcome caused by the semblance of superiority. This ethical paradox requires an open discussion with all stakeholders involved and relentless moral contemplation of designers, architects, and decision makers.

## 6. Limitations

Although investigating the intent to change one’s health behavior, the consequent behavioral changes could not be tracked due to reasons concerning the method. Nor could any differences in therapeutic outcomes be analyzed in this study. Both parameters appear to be important aspects in order to understand the effect the clinic design has on patient’s health outcomes.

The study is limited to patients with orthopedic and rheumatic conditions. Therefore, the sample does not reflect the general public (e.g., average age was 50–59). Hence, the same intervention could lead to different outcomes with patients suffering from other illnesses. The same applies to people at a different age or with a different cultural background.

## Figures and Tables

**Table 1:** Distribution of positive and negative remarks concerning the intention to change one’s health behavior due to rehabilitative treatment.

Group	Intention	No Intention
Pre	146	43
Post	160	26

**Table 2:** Model fit summary and estimates of path analysis.

Dependent Variable		Independent Variable	Estimates	S.E.	C.R.	P
Atmosphere	<--	Intervention	−0.284	0.077	−3.683	0.000
Therapy/Courses	<--	Intervention	0.064	0.051	1.253	0.21
Therapy/Courses	<--	Atmosphere	0.261	0.026	10.101	0.000
Change of health behavior	<--	Intervention	−0.054	0.032	−1.668	0.095
Change of health behavior	<--	Therapy/Courses	0.108	0.025	4.277	0.000
Change of health behavior	<--	Atmosphere	0.04	0.018	2.3	0.021
**Model Fit Summary**						
CMIN			0			
NFI			1			
CFI			1			
RMSEA			0.15			

## Supplementary Materials

The following are available online at www.mdpi.com/2076-328X/7/4/77/s1, File S1: PDF of the questionnaire that has been used (German language).
