# Supplementary material for: Clinic Design as Placebo—Using Design to Promote Healing and Support Treatments
Source: Behav Sci (Basel). 2017 Nov 9;7(4):77. doi: 10.3390/bs7040077 (PMC5746686; doi:10.3390/bs7040077)
Supplement: Supplementary file 1 [file behavsci-07-00077-s001.pdf]

Patienten-Fragebogen zur Acura-Klinik Bad Kreuznach

E4-EnZa2015

Sehr geehrte Untersuchungsteilnehmer,

folgende Umfrage dient der Erforschung zur Gestaltung und Qualitätsverbesserung der Klinik, die im Rahmen einer Forschungsarbeit der Hochschule Darmstadt und Bauhaus Universität Weimar erstellt wird.

Selbstverständlich werden Ihre Angaben vollkommen anonym gesammelt und ausgewertet. Es werden keine personenbezogenen Daten erhoben und keine Daten an Dritte außerhalb der Forschung weitergegeben.

1.) Waren Sie bereits ein früheres Mal in der Acura-Klinik bzw. der Karl Aschoff-Klinik Bad Kreuznach?

☐ Ja, zu Besuch ☐ Ja, zur Behandlung ☐ Nein ☐ keine Angabe

2.) Wie beurteilen Sie im Durchschnitt…

Therapeuten:	sehr gut	☐	☐	☐	☐	☐	sehr schlecht
Ärzte:	sehr gut	☐	☐	☐	☐	☐	sehr schlecht
Pflegepersonal:	sehr gut	☐	☐	☐	☐	☐	sehr schlecht
Sonstiges Personal:	sehr gut	☐	☐	☐	☐	☐	sehr schlecht

3.) Wie beurteilen Sie die Therapie, Übungen und Kurse im Durchschnitt?

sehr gut ☐ ☐ ☐ ☐ ☐ sehr schlecht

4.) Wie beurteilen Sie im Durchschnitt…

Übungs- / Trainings**räume**:	sehr gut	☐	☐	☐	☐	☐	sehr schlecht
Übungs- / Trainings**geräte**:	sehr gut	☐	☐	☐	☐	☐	sehr schlecht
Therapie- / Behandlungsräume:	sehr gut	☐	☐	☐	☐	☐	sehr schlecht
Ihr Zimmer:	sehr gut	☐	☐	☐	☐	☐	sehr schlecht
Warte- und Aufenthaltsräume:	sehr gut	☐	☐	☐	☐	☐	sehr schlecht

---

5.) Wie beurteilen Sie insgesamt die Atmosphäre in der Klinik?

sehr angenehm ☐ ☐ ☐ ☐ ☐ sehr unangenehm

5.b) Was hat hierzu beigetragen?

____

____

6.) Wie beurteilen Sie die Verpflegung in der Klinik?

sehr gut ☐ ☐ ☐ ☐ ☐ sehr schlecht

7.) Wie war die Verständigung bzw. der Zusammenhalt unter den Patienten?

sehr gut ☐ ☐ ☐ ☐ ☐ sehr schlecht

E4-EnZa2015

8.) **Alles in allem, wie beurteilen Sie Ihren Reha-Aufenthalt allgemein?**

sehr gut ☐ ☐ ☐ ☐ ☐ sehr schlecht

8.b) **Warum urteilen Sie so?**

____

____

9.) **Meinen Sie, dass der Reha-Aufenthalt für Sie gesundheitlich nützlich war?**

sehr gut ☐ ☐ ☐ ☐ ☐ sehr schlecht

10.) **Denken Sie, dass der Reha-Aufenthalt Ihr alltägliches „Gesundheitsverhalten“ auf Dauer verändern wird?**

☐ Ja ☐ Nein

10b.) **Wenn JA: Inwiefern?** ______________________________

Wenn NEIN: Warum nicht? ______________________________

11.) **Würden Sie diese Klinik Freunden und Verwandten weiterempfehlen?**

☐ Ja ☐ Nein

12.) **Gibt es etwas, was Sie bezüglich des Klinikaufenthaltes mitteilen möchten?**

____

____

13.) **Alter** ☐ bis 19 ☐ 20-29 ☐ 30-39 ☐ 40-49 ☐ 50-59 ☐ 60-69 ☐ 70-79 ☐ ab 80

14.) **Geschlecht** ☐ m ☐ w

Vielen Dank für Ihre Teilnahme und Unterstützung!
